# Prevalence of obesity and associated risk factors among adults in Kinondoni municipal district, Dar es Salaam Tanzania

**DOI:** 10.1186/1471-2458-11-365

**Published:** 2011-05-23

**Authors:** Grace A Shayo, Ferdinand M Mugusi

**Affiliations:** 1 Department of internal medicine, Muhimbili University of Health and Allied Sciences, P.O.Box 65001 Dar es Salaam Tanzania

## Abstract

**Background:**

Obesity is on the rise worldwide, not sparing developing countries. Both demographic and socio-economic factors play parts in obesity causation. Few surveys have been conducted in Tanzania to determine the magnitude of obesity and its association with these risk factors. This study aimed at determining the prevalence of obesity and its associated risk factors among adults aged 18 - 65 years in Kinondoni municipality, Dar es Salaam, Tanzania from April 2007 to April 2008.

**Methods:**

Random sampling of households was performed. Interviews and anthropometric measurement were carried out to eligible and consenting members of the selected households. Obesity was defined using Body Mass Index (BMI).

**Results:**

Out of 1249 subjects recruited, 814 (65.2%) were females. The overall prevalence of obesity was 19.2% (240/1249). However, obesity was significantly more prevalent in women (24.7%) than men (9%), p < 0.001, among respondents with high socio-economic status (29.2%) as compared to those with medium (14.3%) and low socio-economic status (11.3%), p value for trend < 0.001, and among respondents with light intensity activities (26.0%), p value for trend < 0.001.

**Conclusion:**

This study revealed a higher prevalence of obesity among Kinondoni residents than previously reported in other parts of the country. Independent predictors of obesity in the population studied were increasing age, marriage and cohabitation, high SES, female sex and less vigorous physical activities.

## Background

There are more than 1 billion overweight people (BMI ≥25) in the world [1]. Of those, approximately 350 million are obese (BMI ≥30.0) [1]. Globally, the prevalence of obesity ranges from as low as 0.6% in Gambia among males to as high as 80.2% in Nauru. Among females, obesity ranges from 0.2% in Ethiopia to 78.6% in Nauru [2]. Overall, about 2.5 million deaths are attributed to overweight/obesity worldwide [2,3]. Obesity has been linked to genetic factors as it seems to run families. However, the contribution of environmental factors can not be ruled out in familial obesity. Such families may share dietary and lifestyle habits predisposing to obesity [1]. Environmental factors such as diet and the level of physical activity strongly influence obesity [1,4].

It has been shown that prevalence of obesity increases with age. The association of obesity and age can be explained, in part, by a decrease in the degree of physical activity with age in both men and women [5]. On the other hand, a decrease in metabolism with age, particularly in women after menopause is another reported explanation [3]. Indeed, globally, women have higher rates of obesity than men [6]. Other risk factors positively associated with obesity include marriage, high educational level, alcohol use and high socio-economic status [5,7,8].

Few surveys have been conducted in Tanzania to determine the magnitude of obesity and its association with different risk factors [9,10]. One survey found that the prevalence of overweight and obesity among females aged 15-49 years was 18% and 4% respectively [9]. This study aimed at determining the prevalence of obesity and its associated risk factors among adults aged 18 - 65 years in Kinondoni municipality, Dar es Salaam, Tanzania from April 2007 to April 2008.

## Methods

### Study design, setting and population

This was a cross sectional study conducted in 10 of the 27 wards of Kinondoni Municipal district in Dar es Salaam, Tanzania. Kinondoni district was randomly selected from the three municipal districts of the Dar es Salaam city. It is the largest of the districts in Dar es Salaam and includes both urban and peri-urban areas. According to the 2002 National Census, the Kinondoni municipality has a population of 1,088,867 people with a growth rate of 4.1%. It is estimated that 458,149 residents of Kinondoni Municipality are employed in both private and public sectors. Most people living in Kinondoni 435,242 (95%) are employed in the private sector while the rest 22,907 (5%) are employed in the public sector. In addition, 254,527 people are self-employed. The majority of the residents are involved in petty business, fisheries, livestock keeping and agriculture including horticulture. Only 3% of people work in subsistence agriculture in the peri-urban areas [11].

Adult men and women aged 18-65 who had lived in Kinondoni for one year or longer, were eligible to participate in the study.

Participants were recruited from 10 randomly selected wards out of the 27 wards in the district. Two streets from each of the selected wards were randomly selected, from which a further two ten cell leaderships were again randomly selected. A ten cell leadership is a local governmental authority that is comprised of 10 houses per street. Each set of 10 houses is locally governed by one person known as a ten-cell leader. A total of 20 houses per street were visited and eligible members of a household were studied. The average number of participants per household was four (4). Pregnant women, mothers who were less than 2 months post delivery, women who were on hormonal contraception and participants with oedema or wasting syndrome were excluded.

### Interviews and physical examination

A structured questionnaire was used to collect socio-demographic and clinical information including age, sex, parity, marital status, last normal menstrual period, date of last child birth, and level of education. For weight measurement, participants wore only lightweight clothes and no shoes. Weight was recorded to the nearest 0.5 kg. Height was taken using a height measuring rod without shoes and recorded to the nearest centimetre. Height and weight were used to calculate body mass index (BMI) for each individual. A BMI of < 18.5 kg/m^2 ^was considered to be underweight, while normal weight was a BMI of 18.5 to 24.9 kg/m^2^, overweight 25 to 29.9 kg/m^2 ^and obesity a BMI of ≥ 30 kg/m^2 ^[1,4].

Individuals were asked about their alcohol drinking status and this was coded as alcohol drinker or non alcohol drinker.

A physical activity tool was adopted for this study. This tool consists of questions about leisure and occupational activities which are categorized as light, moderate and vigorous activities depending on the energy expenditure for each, known as Metabolic Equivalent of Task, or simply metabolic equivalent (MET). The MET is a physiological concept expressing the energy cost of physical activities as multiples of Resting Metabolic Rate (RMR). RMR is defined as the ratio of metabolic rate (and therefore the rate of energy consumption) during a specific physical activity to a reference rate of metabolic rate at rest, set by convention to 3.5 ml O_2_·kg^-1^·min^-1 ^or equivalently 1 kcal·kg^-1^· h^-1 ^or 4.184 kJ·kg^-1^· h^-1 ^[12]. By convention 1 MET is considered the resting metabolic rate obtained during quiet sitting. MET values of physical activities range from 0.9 (sleeping) to 18 (running at 17.5 km/h) [12]

A common classification of physical activity by MET is:

• Light-intensity activities defined as 1.1 MET to 2.9 MET;

• Moderate-intensity activities defined as 3.0 to 5.9 METs;

• Vigorous-intensity activities defined as 6.0 METs or more [13].

Adequacy of physical activities in this tool took into consideration the type of physical activity, time and number of days spent on physical activities [14].

To estimate socioeconomic status (SES), household income per month, possession of different properties and assets e.g. land, motorcycle, bicycle, car, a television set, house possession, house renting, size of the house rented or owned in terms of the number of rooms were taken into consideration. The quality of the house was assessed based on the quality of the building materials such as grass thatched roof compared to iron sheet and roof tiles. Types of walls and type of floor were also assessed to estimate SES. Only a wife and a husband were considered joint owners of a household. All children were counted as residents in the house. A family house left to children by their late parents was owned to the head of that family and the rest were considered residents in the house.

### Statistical analysis

Data was entered, cleaned, and analyzed using EPI INFO version 3.3.2 and SPSS version 10.0. All categorical variables were analyzed using frequencies. Cross tabulations and Pearson's Chi-square test were used to obtain the associations and strength of relationship between the independent and the dependent variables. SES of respondents was obtained by the use of factor analysis method whereby variables used to assess SES were taken into consideration. These variables were converted into binary variables, their means, frequencies and standard deviations were calculated. Variables with low standard deviation were given a low weight, meaning that this item is owned by almost all the households or not owned by any of the households and thus it has minimal ability to differentiate the SES of the households. Variables with high standard deviation were given a high weight. A Principal Component Analysis (PCA) was then used to derive factor scores for every weighted variable. Variables that had a positive factor score were associated with a high SES while those with a negative factor score were associated with a low SES. These factor scores were analyzed to generate the 3 categories of SES [15]. Logistic regression analysis was used to control for confounding factors. In this model the dependent variable was BMI while the independent variables were factors that showed statistical significance on chi square test and on univariate analysis including; age, sex, marital status, level of education, SES and type of physical activity. A p-value of ≤ 0.05 was considered significant.

### Ethical issues

Ethical clearance was obtained from the Research and Publication Committee of the Muhimbili University of Health and Allied Sciences. Permission to conduct the study was obtained from local authorities from the municipality and household level. Informed consent was obtained from all participants in the study.

## Results

A total of 400 households were selected and from these one thousand three hundreds and one (1301) adults met the inclusion criteria. Forty three people (3.3%) did not consent. We excluded 9 women who did not meet the inclusion criteria-6 pregnant women, 2 women with pueperium and 1 woman on hormonal contraception. None of the respondents had edema or wasting syndrome.

A total of 1249 respondents were interviewed, examined as per study protocol and their data were analyzed. Socio-demographic characteristics are shown in Table 1. More than half of the subjects (59.5%) were 34 years or younger. Moreover, females constituted about two thirds (65.2%) of the study participants.

**Table 1:** Socio-demographic characteristics, Prevalence of obesity by socio-demographic and behavioral characteristics in the study population (N = 1249)

Characteristic/Risk factor	Total number studied (%)	Number Obese	Percentage obese	P-value
**Age groups**				
18-24	330 (26.4)	22	6.7	
25-34	413 (33.1)	65	15.7	
35-44	251 (20.1)	72	28.7	
45-54	144 (11.5)	46	31.9	
55+	111 (8.9)	35	31.5	0.001*
**Sex**				
Male	435 (34.8)	39	9.0	
Female	814 (65.2)	201	24.7	< 0.001
**Marital status**				
Single	421 (33.7)	35	8.3	
Married/Cohabiting	701 (56.1)	165	23.5	
Divorcee	67 (5.4)	20	29.9	
Widow(er)	60 (4.8)	20	33.3	< 0.001
**Education**				
Informal	129 (10.3)	34	26.4	
Primary	831 (66.5)	162	19.5	
Secondary	246 (19.7)	35	14.2	
Post-secondary	43 (3.5)	9	20.9	0.04
**Parity *(females only, N = 814)***				
0	166 (20.4)	15	9.0	
1	175 (21.5)	28	16.0	
2	171 (21.0)	51	29.8	
3	100 (12.3)	28	28.0	
4	60 (7.4)	18	30.0	
5+	142 (17.4)	61	43.0	< 0.001
**Socio-economic status**				
Low	53 (4.2)	6	11.3	
Medium	775 (62.0)	111	14.3	
High	421 (33.7)	123	29.2	< 0.001*
**Type of physical activity**				
Light	192 (15.4)	50	26.0	
Moderate	795 (63.7)	170	21.4	
Vigorous	262 (20.9)	20	7.6	< 0.001*
**Adequacy of physical activities**				
Adequate	1039 (83.2)	194	18.7	
Inadequate	210 (16.8)	46	21.9	0.278
**Alcohol consumption**				
Yes	242 (19.4)	53	21.9	
No	1007 (80.6)	187	18.6	0.238

P values refer to chi squared test for the deference observed between obese and non obese respondents

* P value for trend

**Figure 1:**
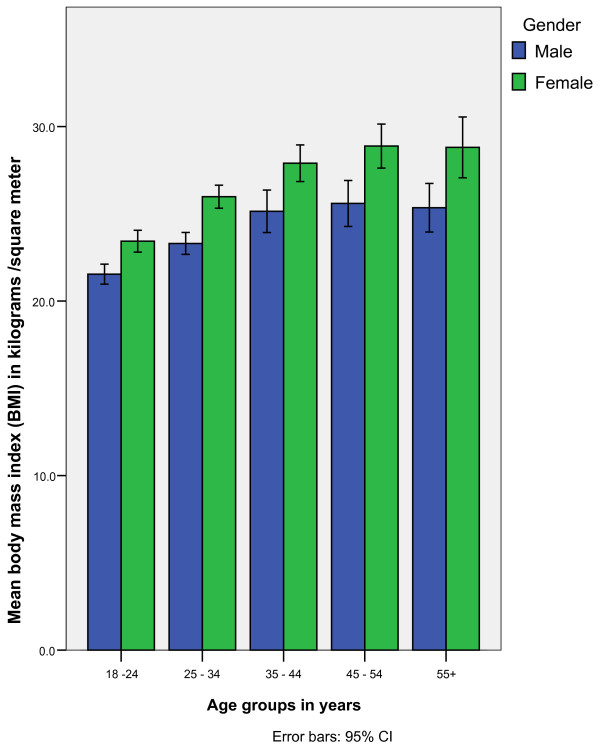
**Increase in BMI by age group in male and female study subjects**.

Obesity was found in 19.2% (240/1249) of participants. Over weight was present in 24.1% (301/1249) of the study participants. Obesity prevalence was highest (31.9%) in age group 45 - 54 years. Age group 18-24 years had obesity prevalence of 6.7%. Age group differences in obesity were statistically significant, p < 0.001 (Table 1). Prevalence of obesity in females was significantly higher than in males (24.7% and 9.0% respectively), p < 0.001. In general, BMI was noted to increase with age, more so in women than men of corresponding age group, p-value for trend < 0.001 (Figure 1). The highest prevalence of obesity (33.3%), was among respondents who were widowed compared to 8.3% among single respondents, p < 0.001 (Table 1).

Prevalence of obesity was significantly higher in participants with no formal education (26.4%) compared to those with primary (19.5%), secondary (14.2%) and post secondary education (20.9%), (p = 0.004) (Table 1). Among females, the prevalence of obesity increased significantly with an increase in parity-9.0% with parity of 0, and 43.0% with parity of 5+, p < 0.001 (Table 1). Sixteen (1.3%) of the respondents had mild under nutrition, that is they presented with mid upper arm circumference (MUAC) of 160 mm - 184.9 mm [16].

The prevalence of obesity was highest among those with high socio-economic status (29.2%) as compared to those with medium (14.3%) and low socio-economic status (11.3%), p < 0.001 for trend. (Table 1)

It was noted that those who did light intensity activities had highest prevalence of obesity (26.0%) followed by those who did moderate intensity activities (21.4%) while those who did vigorous activities had obesity prevalence of 7.6%, (p < 0.001, for trend.), (Table 1).

Prevalence of obesity was higher among alcohol drinkers (21.9%) compared to those who did not drink alcohol (18.6%) but it was not statistically significant, p = 0.238 (Table 1)

In multivariate analysis; increasing age, female sex and vigorous physical activities were independent risks for obesity. Regarding age, the risk was up to five times higher in subjects aged 55 years or older compared to the youngest subjects OR(95% CI) = 5.1(2.5-10.4), p < 0.001. (Table 2) The risk of obesity was 3.6 times higher in females than in males OR (95% CI) = 3.6(2.2-5.4), p < 0.001. (Table 2)

**Table 2:** Socio-demographic and behavioural predictors of obesity by logistic regression among the study population (N = 1249)

RISK FACTOR	UNIVARIATE ANALYSIS OR (95% CI)	P-VALUE	MULTIVARIATE ANALYSIS OR (95% CI)	P-VALUE
**Age groups**				
18-24	1		1	
25-34	2.6(1.6-4.3)	< 0.001	2.3(1.4-3.9)	0.002
35-44	5.6(3.4-9.4)	< 0.001	4.7 (2.6-8.3)	< 0.001
45-54	6.5(3.8-11.5)	< 0.001	5.3 (2.8-10.0)	< 0.001
55+	6.4(3.5-11.6)	< 0.001	5.1 (2.5-10.4)	< 0.001
**Sex**				
Male	1		1	
Female	3.3(2.3-4.8)	< 0.001	3.6 (2.2-5.4)	< 0.001
**Marital status**				
Single	1		1	
Married/Cohabiting	3.4(2.3-5.0)	< 0.001	1.6 (1.0-2.4)	0.054
Divorcee	4.7(2.5-8.8)	< 0.001	1.6 (0.8-3.2)	0.195
Widow(er)	5.5(2.9-10.4)	< 0.001	1.2 (0.6-2.5)	0.670
**Education**				
Informal	1		1	
Primary	0.7(0.4-1.0)	0.073	0.9 (0.6-1.6)	0.923
Secondary	0.5(0.3-0.8)	0.005	0.8 (0.4-1.5)	0.548
Post-secondary	0.7(0.3-1.7)	0.478	1.6 (0.6-4.2)	0.302
**Socioeconomic status**				
Low	1		1	
Medium	1.3 (0.6-3.1)	0.545	1.5 (0.6-3.8)	0.362
High	3.2 (1.3-7.8)	0.009	2.6 (1.0-6.4)	0.053
**Physical activity**				
Light	1		1	
Moderate	0.8 (0.5-1.1)	0.165	0.8 (0.6-1.3)	0.399
Vigorous	0.2 (0.1-0.4)	0.001	0.4 (0.2-0.8)	0.005

Married and cohabiting respondents showed significant increase of the risk for obesity by 60% than were single respondents, OR (95% CI) = 1.6(1.0-2.4), p = 0.054. Widows and widowers had a 20% increased risk for obesity than were single respondents but this was not statistically significant, OR (95% CI) = 1.2(0.6-2.5), p = 0.670 (Table 2). Respondents with high SES showed significant increase of the risk for obesity than were respondents with low SES, OR (95% CI) = 2.6(1.0-6.4), p = 0.053. Respondents who did vigorous activities had a 60% reduction of the risk for obesity as compared to those who did light activities, OR (95% CI) = 0.4(0.2-0.8), p = 0.005. (Table 2)

## Discussion

This study reveals two major findings worthy noting. Firstly the prevalence of obesity is on the rise in Tanzania. Secondly, in the setting of the study population, increasing age, female sex, marriage, high socioeconomic status and less vigorous physical activities increase the likelihood for obesity in the population.

The overall prevalence of general obesity in this study was found to be higher than was previously reported in this country [9,10]. It is prudent to attribute this increase to the increasing urbanization as it was demonstrated in another study conducted in Tanzania that the prevalence of overweight was highest in urban Dar es Salaam than rural Handeni and Monduli[10].

In the present study female sex was associated with an increased risk for obesity. In a study in central Iran, females were more obese than male subjects, an observation attributed to differences in exercise, level of physical activities, and education [7].

In keeping with previously reported studies in Britain and Iran[4,7], in the present study the prevalence of general obesity was found to increase significantly with age. The association can partly be explained by decrease in physical activity [5,17] and decreased metabolism that accompanies aging [3].

In the present study respondents with high socioeconomic status had statistically significant increased risk for obesity than were respondents with low socioeconomic status after controlling for other factors. High socioeconomic status was found to influence weight gain in other studies [5-7]. Reasons given to explain this association in those studies were the relationship between high SES and increased food intake as well as reduced physical activity because of more sedentary lifestyles. This might be the case in the present study.

Findings in the present study showed that those with no formal education had the highest prevalence of obesity. One would expect low socioeconomic status in those with no formal education and thus low prevalence of obesity among those with no formal education. A possible explanation is that in the Kinondoni district about 60% of residents are unemployed and are engaged in self employment, petty businesses, fishing e.t.c. Those who are self employed may have a better income than the employed ones who are far better educated due to the existing low salaries among civil servants in Tanzanian government.

Interestingly, level of physical activity was not a protective factor for obesity, but rather findings suggest that intensity of physical activities was protective. It was found in multivariate analysis that those who did vigorous activities had 60% decreased risk for obesity. This can be explained by the fact that vigorous activities consume more METs than it is for other activities. However, the physical activity tool used in this study could have been either less sensitive or inapplicable to the study population studied; this was a limitation of the study. For example fetching water was equated to weight lifting, dusting or vacuuming was equated to simple house dusting and mopping. The tool is comprised of both leisure and occupational physical activities but in the population studied occupational activities were more common than leisure activities. In addition, most of the participants worked throughout the week for many hours in a day. The majority of participants meet and surpass the 30 - 60 minutes of recommended daily physical activity and thus classified as having adequate physical activities.

Contrary to other researchers, we did not find relationship between alcohol and obesity [18-20]. Lack of association in the present study may be explained by the small proportion of alcohol drinkers in this study.

## Conclusions

This study revealed a high prevalence of obesity among Kinondoni residents which was far higher than previous prevalence obtained from other areas in the country. This means that the obesity epidemic declared in the world by the World Health Organization (WHO) does not spare developing countries where this study was conducted. Independent predictors of obesity in the population studied were increasing age, marriage and cohabitation, high SES, female sex and less vigorous physical activities.

## Limitations of the study

Participants were interviewed at their homes from Mondays to Sundays, from 8:00 am to 6:00 pm. Oftentimes men were not at home during these work hours, thus about two thirds of the study participants were females. This might have skewed the prevalence of obesity as women tend to have higher rates of obesity than men. In addition, the physical activity tool was not validated for the study population, and may have under or overestimated the main findings.

## Competing interests

The authors declare that they have no competing interests.

## Authors' contributions

GAS designed the study, supervised interviews, data collection and entered and analysed the data. FM participated in designing the study and data analysis. Finally, all authors participated in preparation of the manuscript and approved the final manuscript.

## Pre-publication history

The pre-publication history for this paper can be accessed here:

http://www.biomedcentral.com/1471-2458/11/365/prepub
